# Enhancing Neuroplasticity via vagus nerve stimulation to improve urinary dysfunction after spinal cord injury: a perspective

**DOI:** 10.1186/s42234-025-00178-5

**Published:** 2025-06-21

**Authors:** Mia J. Sargusingh, Juliet J. A. Addo, Margot S. Damaser, Philippe Zimmern, Seth A. Hays, Ana G. Hernandez-Reynoso

**Affiliations:** 1 Department of Biomedical Engineering, Case Western Reserve University, 10900 Euclid Ave, Cleveland, OH 44106 USA; 2 Department of Bioengineering, The University of Texas at Dallas, 800 W Campbell Rd, Richardson, TX 75080 USA; 3 Texas Biomedical Device Center, The University of Texas at Dallas, 800 W Campbell Rd, Richardson, TX 75080 USA; 4 Department of Biomedical Engineering, Lerner Research Institute, Cleveland Clinic, 9500 Euclid Ave, Cleveland, OH ND2044195 USA; 5 Glickman Urological and Kidney Institute, Cleveland Clinic, 9500 Euclid Ave, Cleveland, OH ND2044195 USA; 6 Advanced Platform Technology Center, Louis Stokes Cleveland VA Medical Center, 10701 East Blvd, Cleveland, OH 44106 USA; 7 Department of Urology, University of Texas Southwestern Medical Center, 5323 Harry Hines Blvd, Dallas, TX 75390 USA

**Keywords:** Vagus nerve stimulation, Spinal cord injury, Neurogenic bladder, Neuroplasticity

## Abstract

One problematic and undertreated consequence of spinal cord injury (SCI) is urinary dysfunction. Treatment is usually conservative, involving regulation of fluid intake and scheduled bladder emptying through intermittent catheterization. These interventions provide symptomatic relief but are associated with recurrent urinary tract infections and increased risk of kidney disease. Neuromodulation has been used to counteract aberrant signals, such as bladder overactivity, but has yet to address other symptoms, such as urethral sphincter tonic activity or poor bladder compliance. Combining rehabilitation with vagus nerve stimulation (VNS), which is known to engage neuromodulatory nuclei to promote synaptic neuroplasticity and recovery, has emerged as a potential therapy to restore function after neurological injury including SCI. Our perspective is that a congruent strategy of pairing VNS with bladder function after incomplete SCI can promote neuroplastic changes in spared neural pathways to strengthen neural control of bladder function.

## Background

Spinal cord injury (SCI) has an incidence of approximately 18,000 traumatic cases per year in the United States (a Glance. 2024) and approximately 768,000 cases globally (Kumar et al. 2018). Symptoms of SCI can vary according to multiple factors, including the severity and level of the injury (Hamid et al. 2018). The severity of the injury can be classified as complete or incomplete based on the spared motor and sensory function, with approximately 67% categorized as incomplete (Kumar et al. 2018). Incomplete SCI usually spares some ascending sensory and descending motor pathways while interrupting others. Incomplete SCI results in varying levels of paralysis and detrimental effects on long-term daily activities. Urinary dysfunction is a common consequence among individuals with SCI, affecting the majority of patients (Hamid et al. 2018). Its prevalence varies based on factors such as sex, lesion level, and completeness of injury. Among men, who represent 78% of SCI patients (Kumar et al. 2018), approximately 69% continued to experience urinary dysfunction at least one year after injury (Pavese et al. 2016). Among women with incomplete SCI, who represent about 22% of the SCI population (Kumar et al. 2018), urinary incontinence has been reported in approximately 49% of cases (Elmelund et al. 2018). The widespread prevalence of urinary dysfunction in SCI patients highlights the clinical need for robust treatment options.

Urinary dysfunction is a common consequence in individuals with SCI at any level above S1 (suprasacral) despite intact supraspinal and sacral neural pathways. The supraspinal pathways, composed of the pontine micturition center, periaqueductal gray, parahippocampal complex, and medial prefrontal cortex, amongst other neural centers, process sensory input from the bladder and coordinate the continence mechanism and the initiation of voiding (Griffiths 2015). The sacral pathways control the reflexive and voluntary aspects of voiding by facilitating contraction of the detrusor muscle and relaxation of the urethral sphincters. However, after SCI, the neuronal pathways between the supraspinal and sacral neurons are injured, resulting in detrusor-sphincter dyssynergia (DSD), which is characterized by neurogenic detrusor overactivity and non-relaxation of the external urethral sphincter (EUS) (Hamid et al. 2018; Perez et al. 2022; Ikeda n.d.; Groat et al. 2015). Consequently, SCI patients experience a variety of symptoms which decrease their quality of life, including urinary retention, recurrent urinary tract infections, and overflow incontinence (Hamid et al. 2018; Perez et al. 2022). Additionally, high bladder pressures upon storage and voiding increase the risk of vesico-ureteral reflux, irreversible bladder wall changes, autonomic dysreflexia, and renal damage (Hamid et al. 2018).

Therefore, individuals with SCI have routinely ranked restoration of bladder function among their top priorities (Simpson et al. 2012; Anderson 2004). However, there are no consistently effective therapies for chronic urinary dysfunction after SCI. Current approaches provide some symptomatic relief and include behavioral modifications for bladder retraining, fluid intake management, intermittent catheterization, drug therapies, and neuromodulation. But these therapies generally fail to address the core dysfunction which is the disrupted central neural control of bladder circuits (Wyndaele et al. 2010; Ginsberg et al. 2021; Wyndaele 2016). Thus, developing novel therapies to improve and restore bladder function after SCI is critical. In this article, we provide a summary of the currently available management for chronic SCI (including conservative and neuromodulation approaches), followed by a perspective on a new therapy using vagus nerve stimulation (VNS) – an intervention that has been shown to drive targeted neuroplasticity when paired with rehabilitation to reinforce spared neural pathways (Darrow et al. 2020; Meyers et al. 2018; Hays 2016) – to improve the symptoms of bladder dysfunction.

### Available management for chronic SCI

The management of urinary dysfunction after SCI focuses on decreasing risk factors associated with renal damage from elevated pressures in the bladder and/or chronic urinary tract infections (UTIs), which are known to increase mortality risks (Wyndaele 2016; Sekido et al. 2020). The gold standard of care includes conservative management such as controlled fluid intake and timed voiding, with or without pharmacotherapy (e.g., antimuscarinics) to decrease high-pressure urine storage (Wyndaele et al. 2010; Dumoulin, et al. 2017). For patients with a neurogenic overactive bladder, particularly those unresponsive to oral medications, OnabotulinumtoxinA injections into the detrusor muscle may improve bladder capacity and reduce incontinence (Perez et al. 2022; Wyndaele et al. 2010; Ginsberg et al. 2021; Dumoulin, et al. 2017). However, they are associated with a 20.5% (Ginsberg et al. 2021) increase in the risk of urinary retention, often requiring clean intermittent catheterization (CIC) (Perez et al. 2022; Ginsberg et al. 2021). In about 33% of incomplete lesions, specifically in individuals with D or E classification on the ASIA (American Spinal Injury Association) Impairment Scale, bladder emptying may be achieved spontaneously (Patki et al. 2006); however, more than 70% (Zlatev et al. 2016; Joshi et al. 2022; Velaer et al. 2021) of cases require CIC (Zlatev et al. 2016). CIC is preferred over indwelling catheterization (transurethral or suprapubic) which is linked with higher rates of urinary tract infections, bladder stone formation, hematuria, leakage alongside the catheter, catheter clogging, and urosepsis (Ginsberg et al. 2021; Joshi et al. 2022; Chabungbam et al. 2023). However, approximately one-third of cases report difficulties and complications associated with CIC, including time burden, urethral trauma, and recurrent urinary tract infections linked to an increase in mortality rate by approximately 15% (Joshi et al. 2022; Velaer et al. 2021; Wyndaele 2002; Salameh et al. 2015). Emerging or deteriorating renal damage, urinary dysfunction, and chronic or recurrent UTIs are indications that may require surgical interventions (Ginsberg et al. 2021), including sphincterotomy, bladder neck closure with concomitant suprapubic catheter drainage, augmentation cystoplasty, catheterizable continent diversion, or urostomy. These interventions are intended to address specific symptoms – such as neurogenic bladder overactivity or low bladder capacity –only in a small subset of patients who fail conventional therapies or have suffered long-term bladder and/or renal damage (Ginsberg et al. 2021). Because of these SCI management challenges, the use of advanced therapies such as electrical neuromodulation has gained significant interest.

### Neuromodulation therapies

Neuromodulation has emerged as a potential strategy for addressing urinary dysfunction after SCI. An example of this is sacral anterior root stimulation (SARS), which was first established by Giles Brindley in the 1970s and targeted the anterior roots of the sacral nerves S1-S5, because its ramifications innervate the bladder and sphincters through the hypogastric, pelvic, and pudendal nerves, potentially allowing a direct control of lower urinary tract function (Lombardi et al. 2014; Kuris et al. 2022). This technology is capable of bladder emptying or closure of the external EUS on demand (Brindley 1977); however, implantation of this device necessitates a laminectomy for subdural implantation of the electrodes, splitting the strands of the anterior roots. In addition, it may require an irreversible intervention for sensory deafferentation of the sacral posterior roots (rhizotomy) in 75% of SCI patients (Guiho et al. 2022; Ko 2022). Rhizotomy of sacral posterior roots may lead to bladder underactivity and undesired or deteriorating loss of sexual and bowel functions. Because of this, it was only indicated for patients with complete injuries and its use has decreased over the last 40 years due to complications and the rise of other technologies (Guiho et al. 2022). One of these advances was the development of sacral nerve stimulation (SNS) to treat overactive bladder – a urinary dysfunction characterized by frequent and sudden urges to urinate (Lombardi et al. 2014; Guiho et al. 2022; Siegel et al. 2018). SNS has shown at least 50% efficacy and quality of life improvements in up to 82% of implanted patients at 5 years post-implantation (Siegel et al. 2018). Due to its success, SNS was offered to patients with an areflexic detrusor, as the stimulation of the sacral nerve can cause detrusor contraction(Perez et al. 2022; Lombardi et al. 2014). Results have suggested that this approach may significantly increase bladder capacity, decrease residual urine after voiding, reduce maximum bladder pressure, improve urinary continence, and reduce episodes of UTIs (Guiho et al. 2022). However, due to insufficient supporting evidence and a high rate of revision surgeries, the American Urological Association and the Society of Urodynamics, Female Pelvic Medicine & Urogenital Reconstruction have recommended against offering sacral neuromodulation to patients with neurogenic lower urinary tract dysfunction after spinal cord injury (Ginsberg et al. 2021).

Transcutaneous tibial nerve stimulation is another, less invasive, neuromodulation approach being investigated to address bladder overactivity and urinary dysfunction after SCI. Electrical neuromodulation of the afferent posterior tibial nerve may activate the spinal micturition center, competing with neural signals that drive detrusor overactivity, which in turn could lead to the improvement of bladder function. Recent clinical feasibility studies (Stampas et al. 2019; Stampas 2019) have suggested that short-term transcutaneous tibial nerve stimulation during the acute period after SCI may have a modest effect on slowing down the deterioration of urinary dysfunction symptoms, especially those associated with DSD and bladder capacity. However, its long-term effects and efficacy on chronic SCI remain unknown.

Electrical neuromodulation of the sacral, pudendal, and tibial nerves are believed to either achieve direct control or compete with the aberrant signals leading to overactivity. Electrical stimulation of the lumbosacral spinal cord is another modality that has been investigated for direct control of neural activity to achieve volitional voiding after SCI. Epidural placement of electrodes for spinal cord stimulation (Walter et al. 2018; Herrity et al. 2018) has demonstrated the feasibility of activating autonomic and motor spinal cord circuits to modulate the EUS, detrusor pressure, and voiding efficiency (Herrity et al. 2022; Darrow et al. 2022). One challenge with this approach is the optimization of stimulation parameters and mapping of responses, which is likely to vary from subject-to-subject based on anatomical and placement differences (Steadman and Grill 2020). Additionally, other methods of addressing overactive bladder in SCI patients, such as pudendal nerve stimulation (PNS), have been investigated. PNS in cats with chronic SCI has been shown to modulate bladder capacity by inducing excitatory and inhibitory bladder reflexes, though more research is needed to determine its clinical applicability (Tai et al. 2011). While promising, the challenges, limitations, and unknowns associated with these advanced therapies underscore the potential for additional neuromodulation techniques to continue to address these unmet needs.

## The role of VNS in rehabilitation after neurological injury and disease

The aforementioned strategies target specific pathways for direct control or blocking aberrant signals. However, another potential approach for functional restoration is through rehabilitation and neuroplasticity. Individuals with incomplete SCI have spared neural pathways that can provide a foundation for rehabilitation to provide functional recovery (Raineteau and Schwab 2001; Curt et al. 2008; Onifer et al. 2011). Rehabilitation relies on repeated practice and active patient engagement to drive plasticity of the circuits active during a particular task (Hays SA 2016). However, standard rehabilitative tasks in patients with SCI may not be sufficient on their own (Darrow et al. 2020; Hays 2016; Hays et al. 2014a; Khodaparast 2013; Khodaparast et al. 2014; Dawson et al. 2021); neuromodulation interventions may be used to harness neuroplasticity to enhance rehabilitation results. For example, VNS has been demonstrated to promote the release of neuromodulators such as acetylcholine, norepinephrine, and serotonin, throughout the cortex (Follesa et al. 2007; Hulsey et al. 2019; Hulsey et al. 2016). This release is achieved through the activation of neuromodulatory nuclei in the brainstem, including the nucleus basalis, locus coeruleus, and dorsal raphe nucleus, via afferent projections of the vagus nerve (Cunningham et al. 2008; Hulsey et al. 2017). The neuromodulators released by VNS provides a reinforcement signal that strengthens the recently active neural pathways(Meyers et al. 2018; Meyers et al. 2019; Hays et al. 2023). By pairing VNS with a rehabilitative task, the release of neuromodulators is timed with neural activity, thus promoting effective and targeted plasticity in the networks involved in rehabilitation, leading to enhanced recovery(Darrow et al. 2020; Hays et al. 2014a; Khodaparast 2013; Khodaparast et al. 2014; Dawson et al. 2021; Hulsey et al. 2017; Seol et al. 2007; Ganzer et al. 2018).

Preclinical studies in rats have repeatedly confirmed the ability of VNS paired with successful rehabilitative therapy to significantly improve the recovery of motor function in the forelimb in models of stroke and spinal cord injury compared to rehabilitation alone(Darrow et al. 2020; Meyers et al. 2018; Hays et al. 2014a; Khodaparast 2013; Khodaparast et al. 2014; Ganzer et al. 2018). This enhancement of recovery was subserved by synaptic plasticity in motor networks controlling the forelimb. In some cases, functional improvements as a result of VNS-paired rehabilitation were observed via the recruitment of similar muscle activation and neurological pathways that were recruited during the VNS-paired training (Meyers et al. 2018).

This therapy has been evaluated in clinical trials to restore arm and hand functions. A pivotal study (NCT03131960) demonstrated that combining VNS with upper limb rehabilitation significantly improved recovery of arm and hand functions in individuals with chronic stroke and led to FDA approval of this approach (Dawson et al. 2021). In parallel, results for a separate clinical trial (NCT04288245) and preliminary results from an ongoing follow-on sudy (NCT06351111) to restore upper limb function after SCI at the cervical level supported the use of VNS paired with rehabilitation to restore function after injury (Kilgard et al. 2025). Based on these clinical successes and the concept that VNS can drive targeted neuroplasticity when paired with rehabilitation, we hypothesize that a congruent strategy of pairing VNS with bladder function may improve the symptoms of urinary dysfunction after SCI.

### Perspective: Pairing VNS with specific phases of the bladder cycle to improve urinary dysfunction after spinal cord injury

**Fig. 1:**
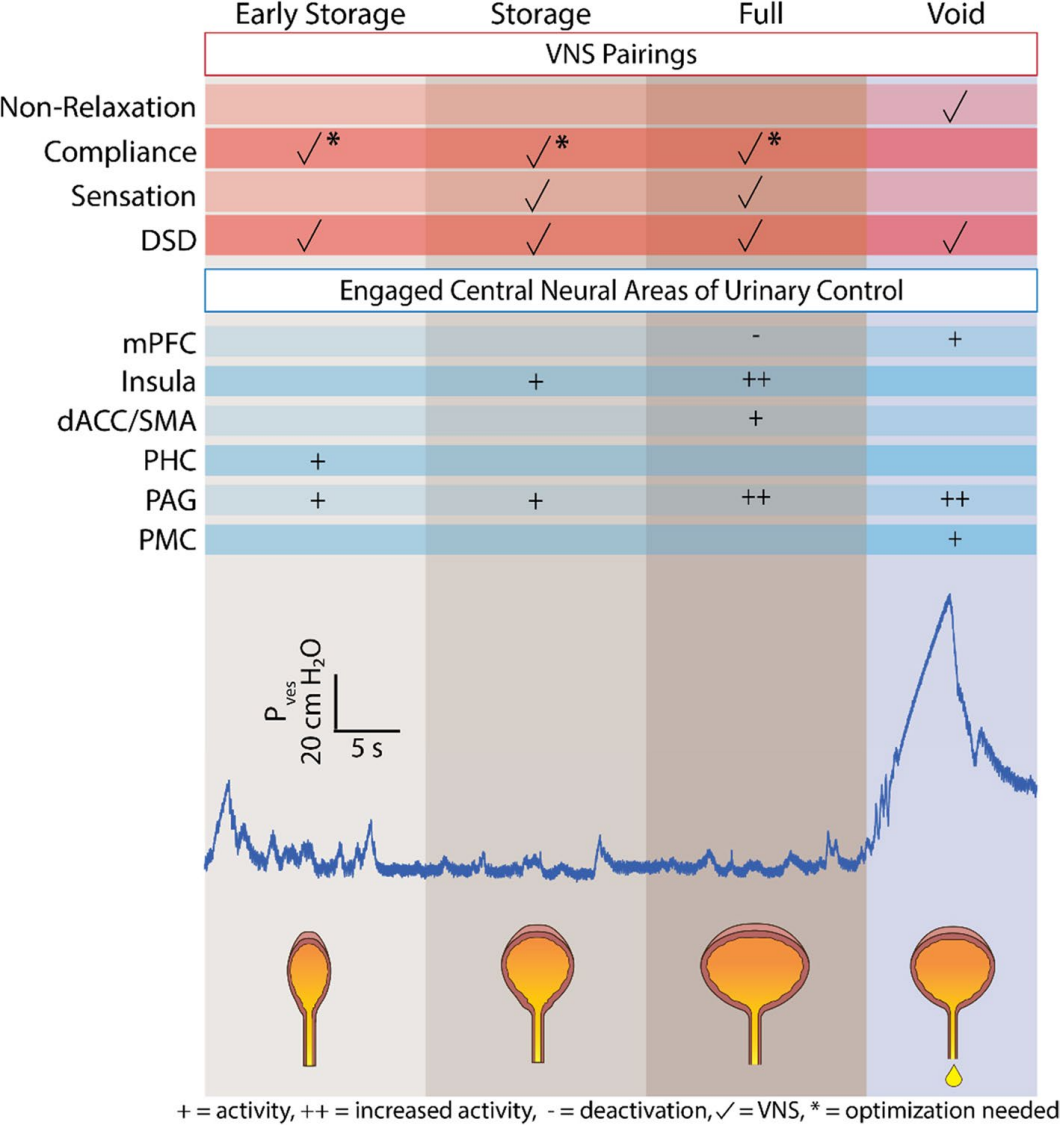
Pairing VNS at different stages of the bladder cycle to drive plasticity in the corresponding engaged neural pathways. During the early storage phase, VNS may reinforce circuits involving the PHC and PAG to improve bladder compliance. In both the storage and late storage/full phase, VNS may improve bladder compliance and sensation but are mediated by promoting different neural circuits. During the storage phase, VNS may promote activity in the insula and PAG, whereas during the late storage/full bladder phase, additional regions such as the dACC, SMA, and mPFC may be enhanced to further improve bladder capacity, compliance, and sensation. Pairing VNS with voiding may enhance pathways involving the PMC and mPFC to address the non-relaxation of the EUS. Optimization may be required to counteract DSD and may require pairing during various phases of the bladder cycle. The cystometrogram trace depicts vesical pressure (P_ves_) recorded in an awake, restrained female Sprague Dawley rat model with an intact spinal cord during retrograde bladder filling at 5 mL/hour via a transurethral catheter. This trace does not represent simultaneous VNS delivery

After an incomplete SCI, some neural pathways that control the bladder may remain intact, but communication between the central nervous system and the lower urinary tract is often dysfunctional. Pairing VNS to evoke neuromodulator release during different bladder function phases may enhance neuroplasticity to increase the cortical representation of neural circuits responsible for bladder storage, and voiding to compensate for lost pathways. This may enable intact pathways to compensate for dysfunctional ones, aiding in the recovery and restoration of bladder function. We hypothesize that pairing VNS with each distinct phase of bladder function may be required to improve specific bladder dysfunction symptoms, as each phase engages different neural circuits (Griffiths 2015; Groat et al. 2015) (Fig. 1). After suprasacral SCI, these circuits become disrupted, resulting in neurogenic lower urinary tract dysfunction. We have identified four key symptoms as potential candidates for restoration via paired VNS therapy: non-relaxation of the EUS (EUS tonic activity), low bladder compliance during urinary storage resulting in high bladder pressure, loss of bladder sensation during filling, and a combination of these neurogenic factors, resulting in DSD.

#### Targeted neuroplasticity vis VNS for non-relaxation of the EUS

The mechanism for pairing VNS with rehabilitation is dependent on the timing of the task and the stimulation delivery (Hays et al. 2014b). The medial prefrontal cortex is typically activated during the voiding phase, suggesting its role in deciding when it is appropriate to void (Weld and Dmochowski 2000; Shy et al. 2014). Neural signals from the medial prefrontal cortex are sent to the periaqueductal gray region of the midbrain and then to the pontine micturition center. This process modulates voluntary relaxation of the EUS by activating the pudendal nerve through the S2-S4 segments of the spinal cord (Perez et al. 2022). Thus, pairing VNS with spontaneous voiding, either simultaneously (Hays et al. 2014b) or within 1–5 s (Ganzer et al. 2018), could target non-relaxation of the EUS (Fig. 1; Void). To achieve this in preclinical investigations with rats after SCI, pairing VNS with spontaneous voiding may be achieved during awake retrograde filling of the bladder by monitoring bladder pressure and predicting an upcoming void based on a thresholding and/or signal processing algorithm or using machine learning approaches (Zareen et al. 2024; Karam et al. 2016). For clinical translation, this pairing may be achieved by manually triggering VNS upon volitional initiation of voiding even if inefficient due to SCI either during the natural voiding cycle with a take-home device, or in a clinical setting with pelvic and bladder rehabilitation exercises. With repeated pairings over time, this strategy could gradually increase voided volume and reduce post-void residual, thus improving voiding efficiency.

#### Targeted neuroplasticity via VNS for bladder compliance and sensory restoration

During the filling of the bladder, different neural circuits are engaged. Afferent fibers of the detrusor muscle stretch and convey information regarding bladder volume via the pelvic nerves to the S2-S4 segments of the spinal cord (Perez et al. 2022; Krhut et al. 2014). This neural information is relayed to the periaqueductal gray and parahippocampal cortex while the volume remains low (Groat et al. 2015) (Fig. 1, Early Storage). Then, the pontine micturition center activates the sympathetic hypogastric nerve, which relaxes the detrusor muscle, achieving bladder filling at low pressure (adequate bladder compliance). When the bladder nears maximum capacity, the insula becomes activated which signals the desire to void (Groat et al. 2015; Krhut et al. 2014) (Fig. 1, Storage/Full). Subsequently, the anterior cingulate cortex activates to inhibit bladder contraction, and the supplementary motor cortex co-activates for EUS and pelvic floor muscle contraction, to prevent voiding and maintain the continence mechanism (Groat et al. 2015) (Fig. 1, Full). After suprasacral SCI, the detrusor muscle is unable to relax, resulting in low compliance with urinary storage pressures exceeding 40 cmH_2_O, a threshold reported to provoke bladder wall damage, reflux, and possible renal damage (McGuire 2010). We hypothesize that delivering VNS with filling of the bladder, may reinforce the sympathetic pathway of the periaqueductal gray and parahippocampal cortex, improving detrusor compliance (Fig. 1, Early Storage). However, it is important to note that compliance is multifactorial, influenced by neural signaling as well as fibrosis and chronic inflammation after SCI. At this time, it is unknown whether VNS may play a role in the resolution of fibrosis and inflammation of the bladder, but the effects of VNS may help these conditions through a secondary anti-inflammatory mechanism, rather than neuroplasticity alone (Bonaz et al. 2016). For validation of this approach in the preclinical rat model of SCI, precise optimization of the timing is essential to improve detrusor compliance during awake retrograde filling and voiding. This involves determining whether VNS should be delivered throughout the entire filling phase or would be more effective during specific phases (e.g., early, mid, or late phase of bladder filling). This investigation requires a systematic approach, involving experimental groups for all the VNS pairings described. After optimization, clinical translation of this strategy requires knowledge of bladder volume or pressure, which is usually achieved only in the clinic through invasive urodynamic or video-urodynamic studies. However, the recently reported novel catheter-free ambulatory bladder pressure monitor (Frainey et al. 2023) may be used for estimation of bladder state and could be utilized in combination with an implantable Vagus nerve stimulator to deliver automated, closed-loop, paired VNS at home. Noninvasive bladder volume measurements could likewise be used to estimate phase of filling for triggering VNS at home (Kang et al. 2023).

Developing a pairing strategy for targeted neuroplasticity via VNS is challenging in preclinical models due to limitations on assessing bladder sensory perception. In humans, sensory restoration may be achieved by manually triggering VNS upon the first bladder sensation, when the insula is active, and strong desires to void, usually when the bladder has reached maximum capacity. However, SCI may suppress the sensation of desire to void in some individuals. We cannot exclude the possibility that pairing with filling, especially nearing the desire to void, may improve sensation without the need for additional pairing, but further optimization will be required.

Additionally, given the evidence of SCI-induced neurochemical plasticity in the nodose ganglion (NG), including in bladder-innervating afferents, it is possible that VNS delivered during bladder filling could engage this extraspinal structure directly, promoting plasticity that enhances or restores bladder sensation. Further investigation is needed to determine whether mechanism contributes to improved perception of bladder fullness or urgency following VNS.

#### Targeted neuroplasticity vis VNS for DSD

DSD after SCI is a combination of these non-relaxation of the EUS, high bladder pressures during filling, and loss of bladder sensation; therefore, a strategy of pairing VNS with all three phases (i.e., voiding, filling, and sensation) may harness neuroplasticity of the dysfunctional neural pathways associated with each symptom and restore the coordination needed for effective continence and micturition (Fig. 1). There may be two approaches. First, because it is believed that paired VNS leads to long-term synaptic potentiation (Meyers et al. 2018), a strategy of addressing each symptom individually, one sequentially after the next, may be sufficient. Second, to minimize the burden of rehabilitation on patients, a strategy of pairing VNS with all phases simultaneously may be effective at overcoming DSD.

#### Maximizing the effect of targeted neuroplasticity via VNS

A consideration with SCI is that although the vagus nerve remains anatomically intact, studies have shown that SCI induces neurochemical changes within the NG – the primary sensory ganglion of the vagus nerve. Specifically, studies in rats have shown that SCI leads to upregulation of the nociceptive marker P2X3 and decreased isolectin B4 binding in NG neurons, including afferents projecting to the bladder (Herrity et al. 2015). These changes may alter sensory signaling properties after SCI. Consequently, there is a need for a mechanistic investigation into VNS and bladder function, including synaptic tracers, to fully explore the interaction between peripheral neurochemical changes and targeted plasticity driven by VNS. In future studies, animal research can be leveraged to understand the role of targeted neuroplasticity driven by VNS paired with bladder function. However, a recent study in rats after SCI demonstrated that, despite these potential neurochemical changes, VNS paired with upper limb rehabilitation effectively promoted synaptic reorganization of the motor cortex. This was evidenced by an increased number of labeled neurons connected to forelimb muscles, as per synaptic retrograde tracing, compared to rehabilitation alone (Ganzer et al. 2018). Further mechanistic studies in rats, albeit in contexts other than SCI, have investigated the effect of depleting neuromodulators at the central nervous system level (i.e., acetylcholine, norepinephrine, and serotonin) to prevent an increase in the cortical representation of proximal forelimb, as per intracortical microstimulation mapping (Hulsey et al. 2019, 2016). These studies suggest that targeted neuroplasticity driven by VNS paired with rehabilitation is feasible despite peripheral changes after injury. However, given the complex neural control of bladder function, parametric optimization of paired VNS therapy may be needed to account for peripheral neurochemical and anatomical changes after SCI to ensure efficacy.

Moreover, Ganzer et al. (Ganzer, et al. 2018) demonstrated that conditional pairing of VNS with an adaptive threshold protocol (i.e., changing the threshold to deliver stimulation on movements that better approximate the desired outcome) is significantly better than pairing sub-threshold. This study proposes the thesis that a conditional pairing may be beneficial to drive additional neuroplasticity in a desired direction. For example, when pairing with voiding to overcome non-relaxation of the EUS, pairing only those voids below a certain P_ves_ threshold could decrease voiding pressure, thus decreasing the long-term risk of kidney damage. When pairing with filling, pairing only when the filling pressure is below 40 cmH_2_O could maximize bladder compliance.

Finally, individuals with SCI above the T6 level often experience autonomic dysreflexia – a life-threatening condition characterized by the uncontrolled response of the autonomic nervous system to stimuli, often from bladder overdistention, below the level of the injury (Karlsson 1999). VNS used for targeted plasticity therapy has already been shown in preclinical studies to be safe and does not trigger autonomic dysreflexia, nor does it worsen its severity (Sachdeva et al. 2020). Additionally, improving bladder compliance with urinary storage pressures below 40 cmH_2_O and restoring the desire to void may reduce bladder overdistention, reducing the triggering factors associated with autonomic dysreflexia.

## Conclusion

SCI profoundly disrupts bladder function, negatively affecting quality of life and increasing the risk of renal damage and mortality due to recurrent UTIs, urosepsis, or chronic kidney failure. Current therapeutic approaches generally offer limited relief of symptoms and fail to restore neural control. Our innovative strategy incorporates VNS paired with targeted rehabilitation of bladder function, harnessing neuroplasticity to enhance motor and sensory recovery of lower urinary tract function after SCI. This approach goes beyond symptomatic management, aiming to fundamentally restore the neural pathways associated with bladder function. Previous preclinical studies have already shown that VNS, combined with rehabilitation, markedly improves recovery of upper motor function following neurological damage more effectively than rehabilitation alone. An active follow-on clinical trial (NCT06351111) is exploring paired VNS for restoring motor and sensory arm and hand function after SCI, leveraging the success of similar strategies. VNS offers a new practical strategy to restore bladder function, reduce healthcare costs, and enhance the quality of life of individuals living with SCI, in addition to reducing their risks of devastating and life-threatening urological complications.

## Data Availability

This research project did not involve the generation of new data, except for visualization of bladder pressure in Fig. 1. Data can be made available upon request. All sources of data, literature, and previously published works were properly cited. Analysis and interpretations were conducted avoiding any form of data manipulation or misrepresentation.

## Abbreviations

AIS: American Spinal Cord Injury Association Impairment Score
CIC: Clean Intermittent Catheterization
DSD: Detrusor-Sphincter Dyssynergia
DACC: Dorsal Anterior Cingulate Cortex
EUS: External Urethral Sphincter
mPFC: Medial Prefrontal Cortex
NG: Nodose Ganglion
PAG: Periaqueductal Grey
PHC: Parahippocampal Cortex
PMC: Pontine Micturition Center
PNS: Pudendal Nerve Stimulation
SARS: Sacral Anterior Root Stimulation
SCI: Spinal Cord Injury
SMA: Supplementary Motor Area
SNS: Sacral Nerve Stimulation
UTI: Urinary Tract Infection
VNS: Vagus Nerve Stimulation
